# A case of endocardial dissection caused by Micra implantation

**DOI:** 10.1186/s12872-023-03550-y

**Published:** 2024-01-02

**Authors:** Jianghua Zhang, Shuai Shang, Zhenyu Dong, Xianhui Zhou, Yaodong Li, Yanmei Lu, Qiang Xing, Zukela Tuerhong, Yankai Guo, Jiasuoer Xiaokereti, Baopeng Tang

**Affiliations:** 1 Department of Pacing and Electrophysiology, The First Affiliated Hospital of Xinjiang Medical University, Urumqi, Xinjiang China; 2 Xinjiang Key Laboratory of Cardiac Electrophysiology and Remodeling, The First Affiliated Hospital of Xinjiang Medical University, Urumqi, Xinjiang China

**Keywords:** Micra, Leadless pacemakers, Endocardial tear

## Abstract

**Background:**

Leadless pacemakers are a recent technological advancement. It has many advantages, but there are still a few serious complications.

**Case presentation:**

This article reports the case of a patient with an endocardial tear and dissection caused by contact with the tip of the Micra cup during surgery and summarises the relevant data.

**Conclusions:**

This case report details the occurrence and management of the incident and provides some guidance for future clinical management.

## Introduction

Leadless pacemakers (LPs, Micra, Medtronic, USA) are a recent technological advancement. The main advantage is the absence of leads and therefore a net reduction in all lead-related complications (mainly infections but also long-term fractures). However, in the acute phase of Micra implantation, the risk of cardiac perforation and consequent death is higher than in conventional pacemaker implantation [1, 2]. Previous studies have shown that endocardial injury caused by contact with the cups of the Micra delivery system during implantation may be a risk factor for cardiac perforation [3]. Although the incidence of such a complication is not high, we cannot ignore it because of its high mortality rate. This article reports the case of a patient with an endocardial tear and dissection caused by contact with the tip of the Micra cup during surgery and summarises the relevant data.

## Case presentation

A 77-year-old female (height 148 cm, weight 70 kg, BMI 31.96 kg/m^2^) with a 4-year history of hypertension, who sustained a stroke 2 years ago and regularly took antihypertensive drugs, antiplatelet drugs and lipid-lowering drugs, had repeated transient loss of consciousness. The electrocardiogram showed intermittent third-degree atrioventricular block, and she was admitted to our hospital for further treatment. After the relevant examinations, the diagnosis of high-degree atrioventricular block was confirmed, and we decided to implant a leadless pacemaker (LP, Micra, Medtronic, USA).

**Fig. 1:**
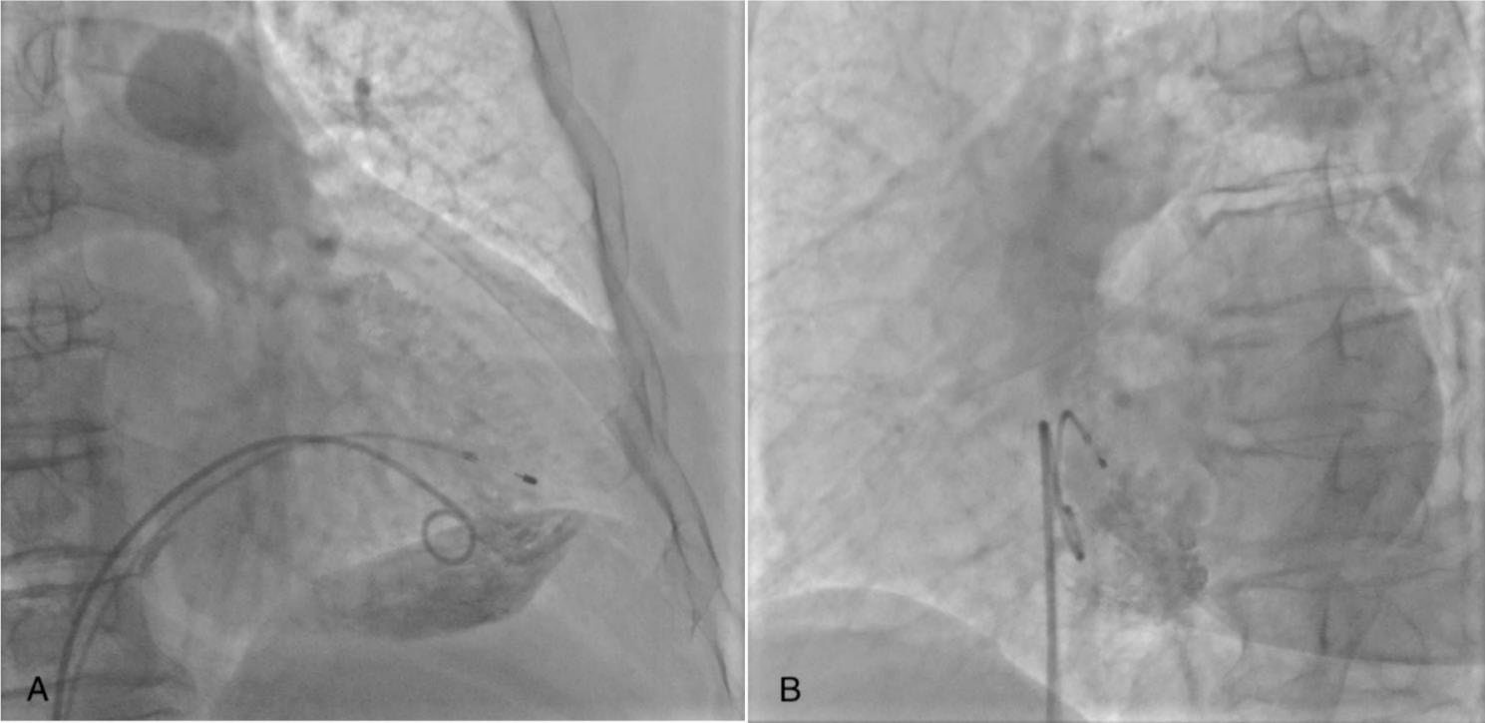
Preoperative right ventriculogram showed an intact endocardium. (A. Right anterior oblique position; B. Left anterior oblique position)

**Fig. 2:**
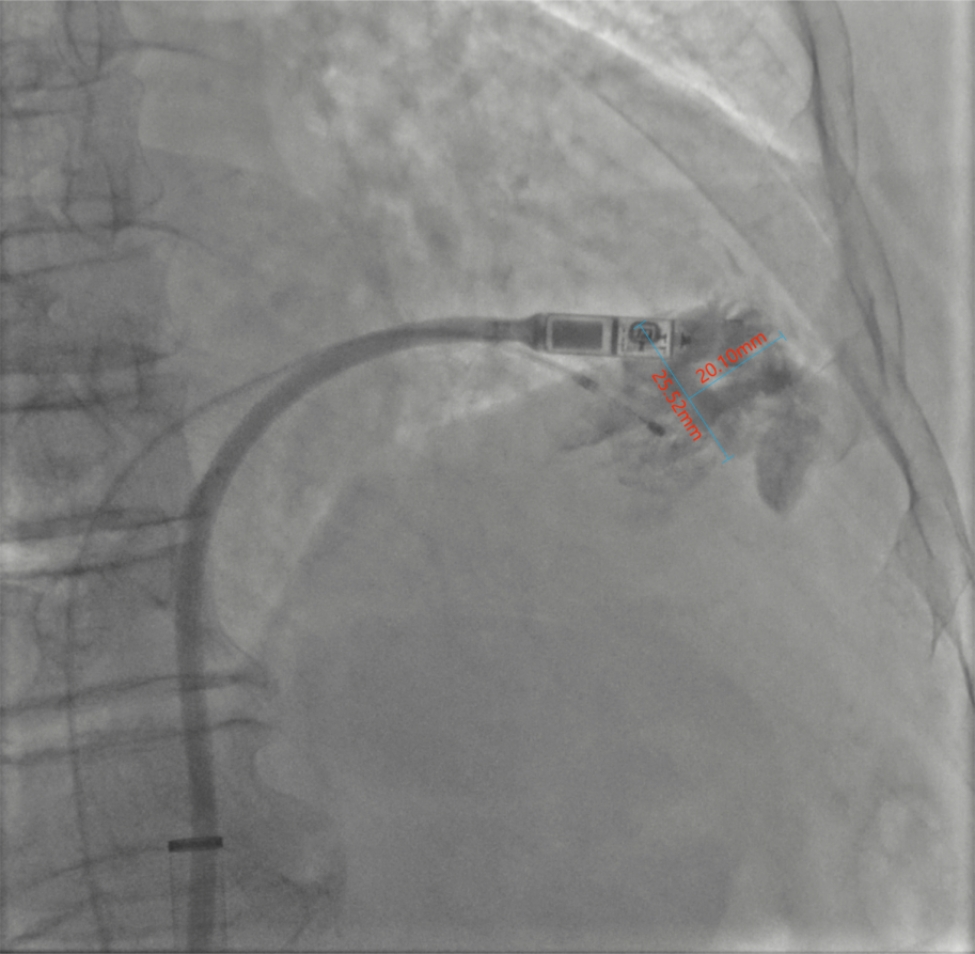
Contrast agent was retained after injection, forming a pocket-type structure with an opening diameter of approximately 25 mm and an axial length of approximately 20 mm

**Fig. 3:**
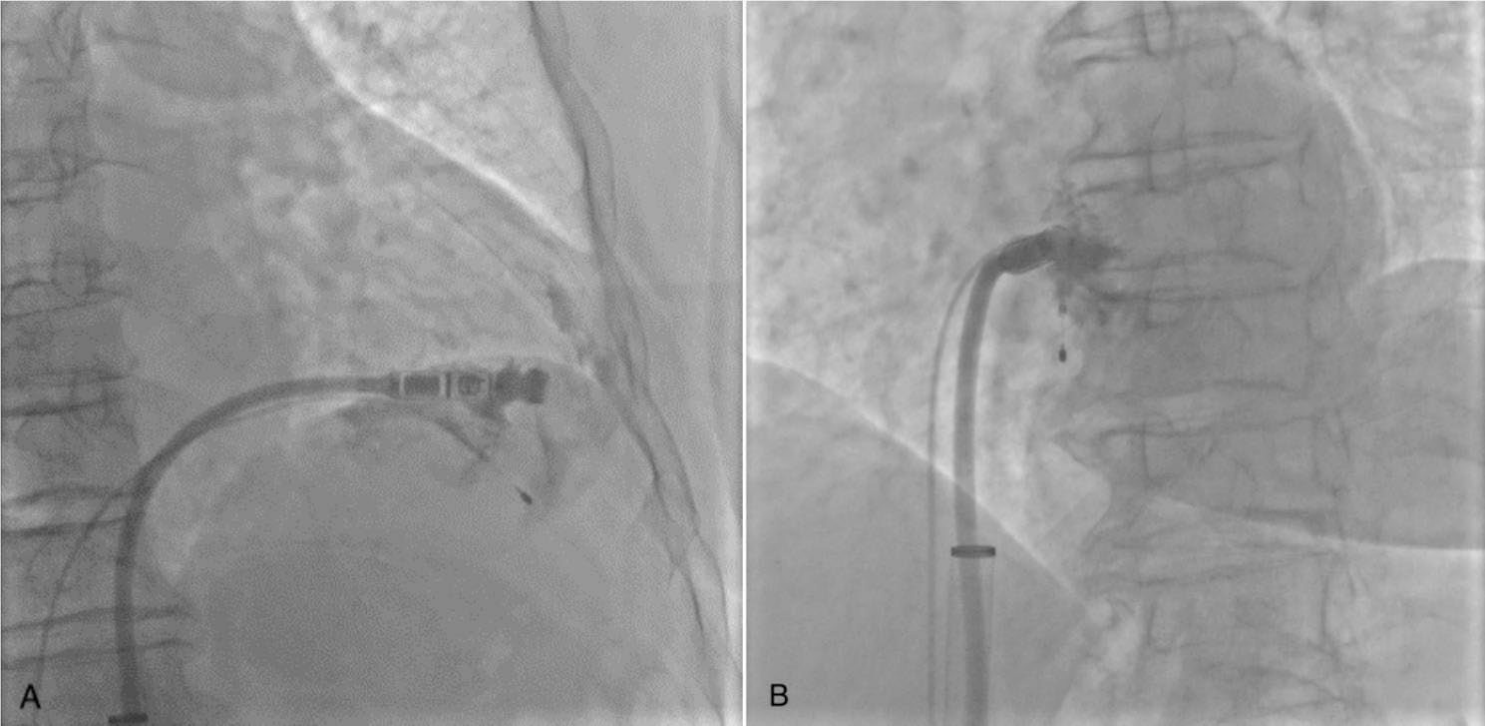
Extravasation of contrast into the pericardial cavity when angiography was performed near the area of residual contrast. (A) Right anterior oblique position; (B) Left anterior oblique position)

Under local anaesthesia, 8-F sheaths were placed percutaneously in the right femoral vein, a super stiff guidewire and pigtail catheter were passed through the sheath to the superior vena cava, and the pigtail catheter was placed in the right ventricle for right ventriculography in the right anterior oblique and left anterior oblique positions, which showed that the endocardium was intact and had no abnormalities (Fig. 1A, B). The delivery catheter was advanced to the right mid-ventricular septum where the device cup was gently positioned after crossing the tricuspid valve. Subsequent right anterior oblique and left anterior oblique views were taken to ensure that the device cup was positioned over the septum, and contrast agent was slowly injected to enhance the visibility of the site where the Micra would be delivered. However, we found residual contrast beyond where the Micra was deployed. The cup was pocket shaped with a large mouth and small tail, and an opening diameter of approximately 25 mm (Fig. 2). Therefore, the head port was adjusted to allow delivery of the Micra to the low septum, and electrical testing was performed, which showed a pacing threshold of 2.0 V/0.24 ms, impedance of 500 Ω, and RV amplitude of 3.5 mV. With unsatisfactory electrical parameters, we repositioned the Micra to the mid-septum, and reimaging showed that the contrast medium was extravasated into the pericardial space (Fig. 3A, B).

**Fig. 4:**
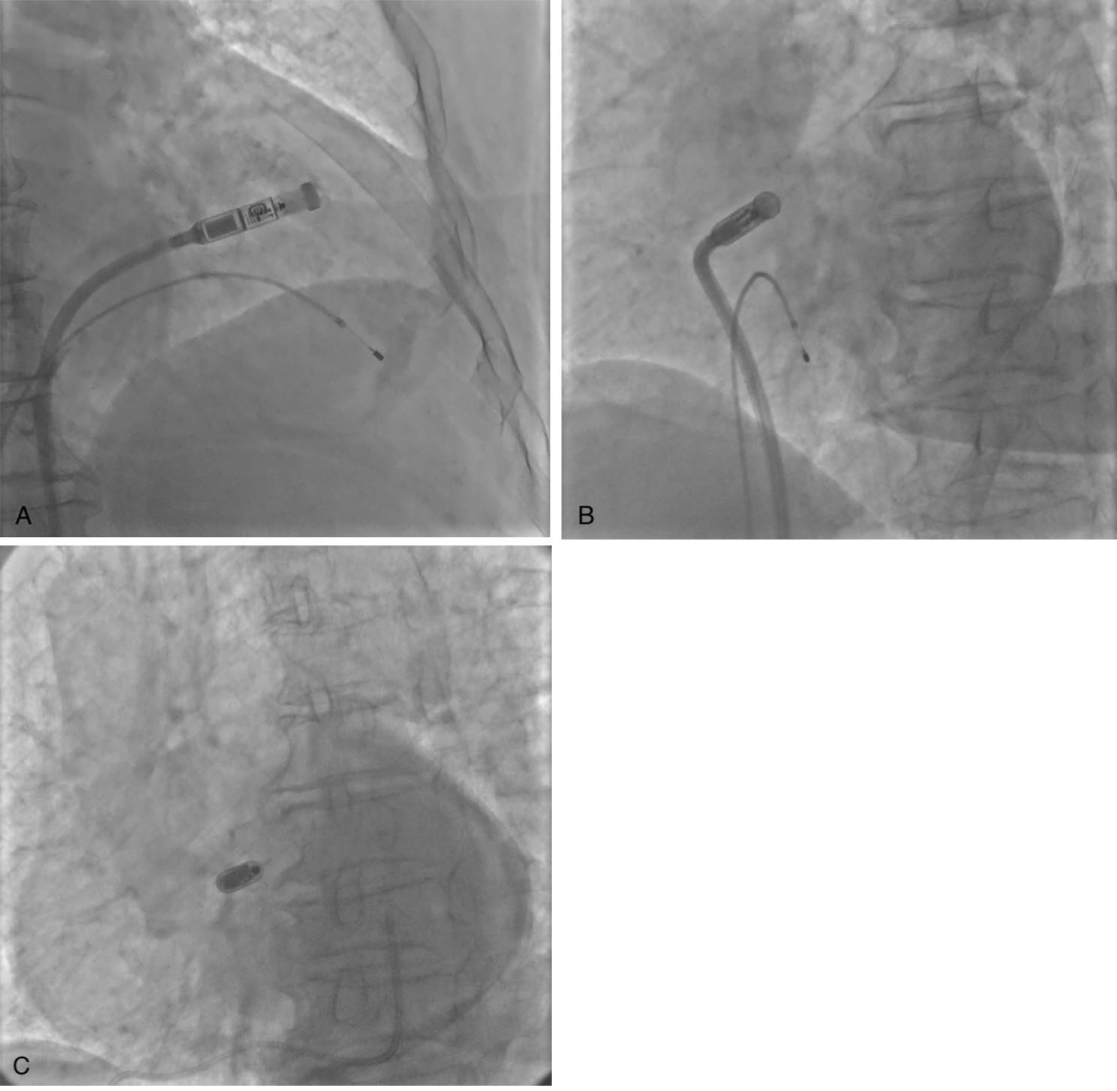
Release the Micra at a high intervals after adjusting the position. (A. Right anterior oblique positioning image; B. Left anterior oblique positioning image; C. Left anterior oblique fluoroscopic Micra final release parallel to the image of the pericardial perforator tube.)

**Fig. 5:**
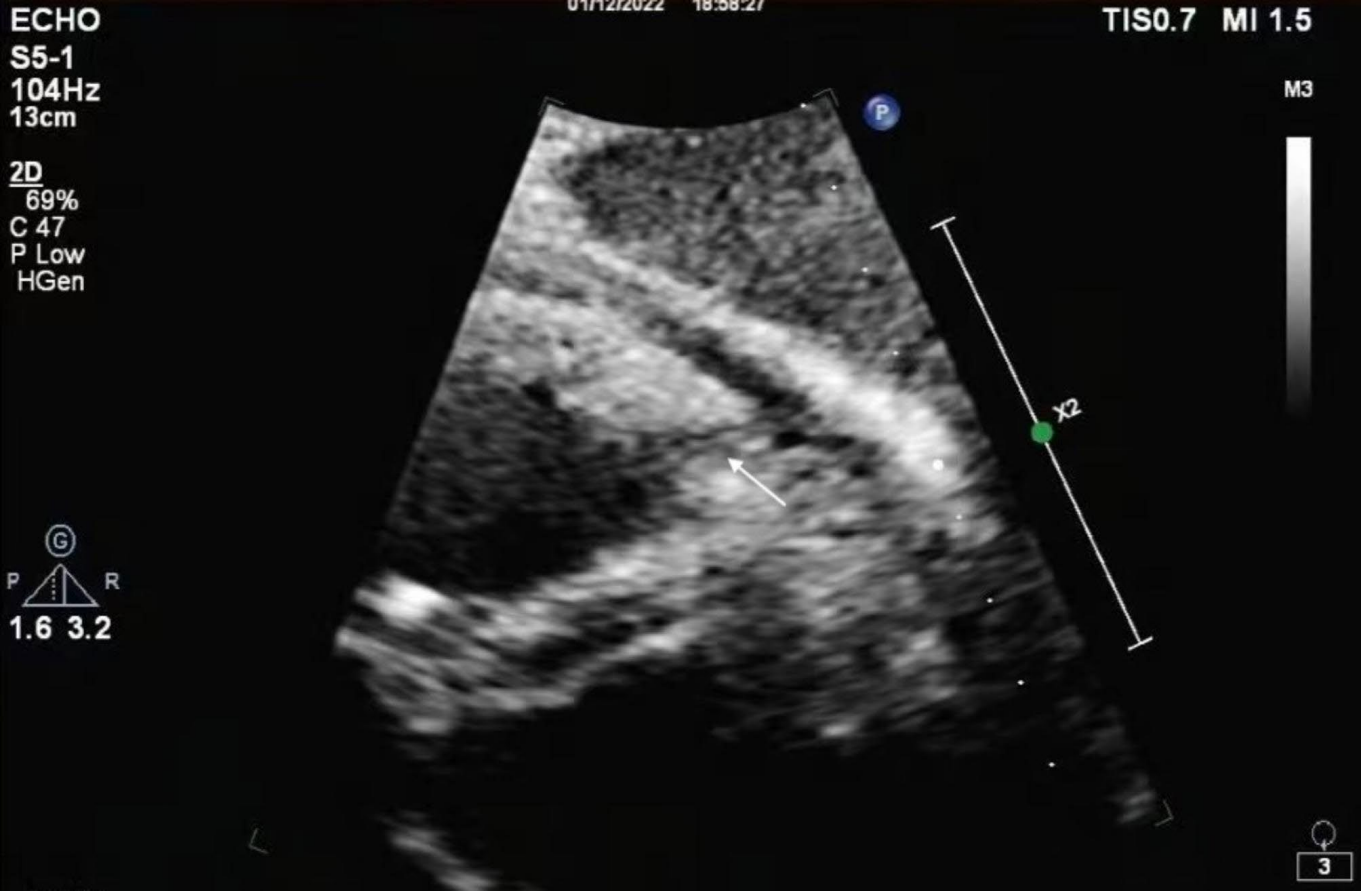
Postoperative cardiac echocardiography revealed an endocardial tear in the apical region of the right ventricle

**Fig. 6:**
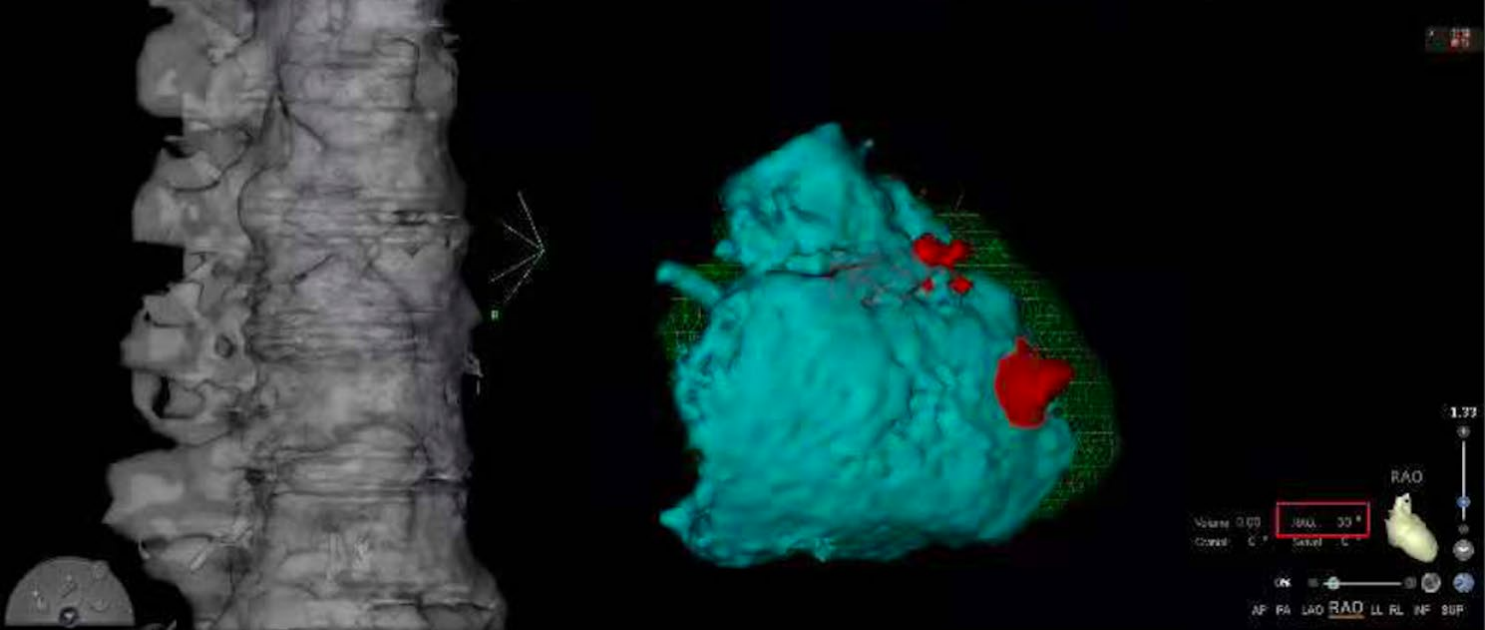
Cardiac CTA was performed 3 days after surgery and showed an endocardial defect in the region of the median septal sulcus of the right ventricle

Her blood pressure remained at 100–110/60–70 mmHg. The Micra was again deployed to the high septum of the right ventricle, ensuring good contact between the delivery system cup and the endocardium of the LAO and RAO (Fig. 4A, B, C). The pacing threshold was 0.88 V/0.24 ms, the impedance was 1080 Ω, and the R-wave amplitude was 7 mV. Approximately 20 min after Micra deployment, the patient’s blood pressure began to fall to 74/59 mmHg, and fluoroscopy showed that her cardiac shadow beat had weakened. With regard to the suspected pericardial tamponade, pericardiocentesis was performed, and approximately 100 ml of bloody pericardial effusion was drained, after which the patient’s vital signs remained stable. Postoperative echocardiography showed a small amount of pericardial effusion and a tear in the endocardium, resulting in dissection (Fig. 5). Three days after implantation, cardiac CTA was performed, which showed a cavity-like structure protruding from the surface of the heart in the right ventricle (Fig. 6). No increase in pericardial effusion volume was noted on continuous echocardiographic monitoring after surgery, and she was discharged 5 days after surgery.

**Fig. 7:**
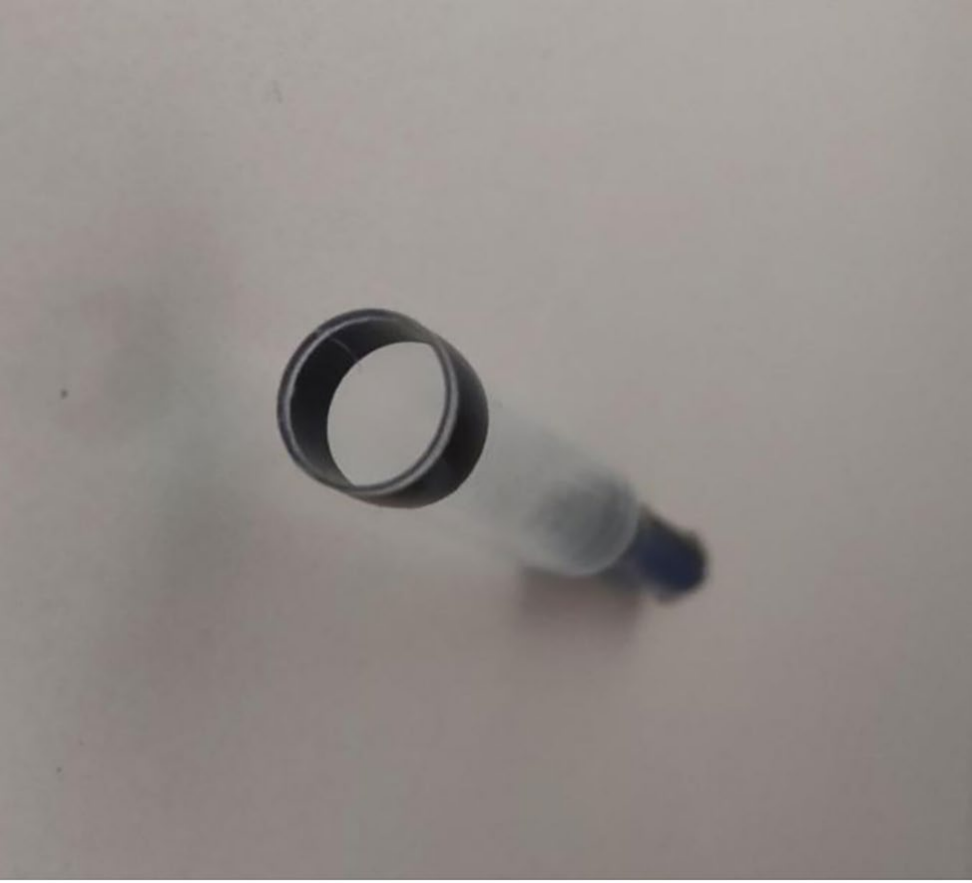
Micra release cup. The visible edge of the cup has an outwardly rolled contour,, but the edge is still relatively sharp considering that it is only 0.3 mm thick

One month after implantation, the patient’s echocardiogram still showed a moderate amount of pericardial effusion of approximately 400 ml, but puncture drainage was not performed. At the 2-month follow-up after surgery, echocardiography showed a large amount of pericardial effusion of approximately 800 ml. The patient was readmitted for pericardiocentesis and drainage. The patient was followed up with monthly echocardiograms for the next six months, and no pericardial effusion was found. Micra parameters and function were normal during the follow-up period.

## Discussion and conclusions

As a new pacing therapy developed in recent years, Micra has many advantages, such as no lead, no pocket, and a small size. However, there is still an adverse event rate of approximately 4%. Vascular complications included cardiac tamponade or perforation (1.6%) and pseudoaneurysm (0.7%) [4, 5]. Among these, cardiac perforation or tamponade is one of the most serious complications as it can lead to patient death. Although the incidence is not high, it is one of Micra’s main safety concerns [6, 7]. Recent studies reported that 563 cardiac perforations occurred within 30 days after implantation, between 2016 and July 2021, and that most perforations were device- and/or operator-related, warranting Micra recapture and causing further endocardial damage due to positioning and deployment, incorrect site of Micra re-implantation, and inappropriate tip pressure during deployment [3]. In one case, the delivery system was close to the free wall when the Micra was implanted so myocardial perforation and acute cardiac tamponade occurred after contrast injection, and the Micra was reimplanted in the septum after pericardiocentesis [8]. Togashi [9] reported a case of asymptomatic cardiac perforation caused by the tip of the cup injuring the free wall of the right ventricle during implantation.

Previous reports have shown that having a low body mass index and a history of comorbid lung disease increase the risk of cardiac perforation during Micra implantation [10]. A recent study of an international registry (i-LEAPER registry) showed that being female (and therefore having a smaller right ventricular chamber) was not associated with an increased risk of adverse events or cardiac perforation during Micra implantation [11]. However, cases of endocardial dissection have not been reported in any studies. The sharp edge of the Micra cup (Fig. 7), which is approximately 0.30 mm, may be the main cause of perforation and endocardial dissection. Despite the external roll treatment, the edge is still relatively sharp without passivation. There is still a risk of cutting the myocardium at a certain angle, especially when the release cup touches the myocardium at a certain pressure. The operator rotates the delivery system clockwise to direct the release cup towards the ventricular septum, which is more likely to cut the endocardial myocardium. We believe this is an important risk factor for endocardial tear. Therefore, the delivery catheter should be rotated clockwise within the right ventricular cavity, taking care to avoid abrupt contact with the myocardium. In the present case, endocardial rupture may have occurred at the edge of the Micra release cup at the time of positioning, and the rapid injection of contrast here caused endocardial tear and dissection due to its impact, resulting in residual contrast appearing here. During postoperative follow-up, the patient was found to have chronic pericardial effusion, which may have been caused by the rupture of the endocardial dissection that did not heal within a short time during surgery.

Analysis of the causes of endocardial dissection in this patient led us to suggest the following improvements: (1) Patients who are elderly have inherent risk factors for endocardial injury. Each patient’s general condition and previous comorbidities should be fully assessed prior to the procedure. (2) The design of the Micra cup is imperfect in that the edge of the cup is thin and sharp and can easily damage the endocardium. We found that the new Micra AV/VR(second generation) catheter tip is 54% thicker with a rounded edge that applies less pressure during deployment. (3) If the surgeon performing the procedure made the goose neck before deployment, appropriate pressure should be applied to avoid perforation, taking care to avoid excessive stress on the myocardium. (4) Intraoperative rotation after the release cup touches the myocardium should be avoided as much as possible to reduce the risk of the release cup cutting the endocardium, while contrast medium should not be pushed too fast during positioning to avoid endocardial dissection due to myocardial tearing. (5) The operator should be aware of residual contrast in the myocardium, which may be a manifestation of endocardial dissection, and should avoid further angiography in the area of residual contrast and should not deploy the Micra in the area of residual contrast. (6) The patient’s vital signs should be closely monitored intraoperatively and postoperatively. If cardiac tamponade occurs, pericardiocentesis should be performed immediately, and cardiac repair surgery should be performed if necessary.

## Data Availability

Not applicable.
